# Biomechanical comparison of palmar plate plus headless compression screw versus radiopalmar double plating in AO/OTA 23-C2.1 distal radius fractures

**DOI:** 10.1007/s00402-026-06279-3

**Published:** 2026-04-06

**Authors:** Patrick Riegner, Christian Spiegel, Felix Christian Kohler, Heike Kielstein, Ivan Zderic, Boyko Gueorguiev-Rüegg, Mark Lenz, Wolfram Weschenfelder

**Affiliations:** 1 Jena University Hospital, Jena, Germany; 2 University Hospital Halle, Halle (Saale), Germany; 3 AO Research Institute Davos, Davos, Switzerland

**Keywords:** Distal radius fracture, Palmar locking plate, Headless compression screw, Radio-palmar double plating

## Abstract

**Introduction:**

This biomechanical study compared the fracture stability of a palmar plate combined with a headless compression screw (HCS) with that of a radiopalmar double-plate construct for AO/OTA 23-C2.1 distal radius fractures with metaphyseal defect zones.

**Materials and Methods:**

Eleven matched pairs of cryopreserved human radii were prepared with standardized AO/OTA 23-C2.1 fractures. Left radii (n = 11) were fixed with a palmar plate plus HCS, while right radii (n = 11) received a radiopalmar double-plate construct. Construct stiffness and axial displacement were assessed using a universal testing machine. Interfragmentary range of motion (ROM) and rotation (ROT) were quantified using an optical three-dimensional motion-tracking system. Measurements were obtained before and after 5000 cycles of dynamic axial loading at 150 N. Two specimen pairs were excluded due to early failure or incomplete data acquisition.

**Results:**

Both constructs demonstrated comparable stiffness and axial displacement, with no implant loosening or hardware failure observed. Interfragmentary ROM did not differ significantly between groups. However, the plate–HCS construct showed greater variability in rotational parameters. Initial radial-shaft rotation was significantly greater in the plate–HCS group (1.14° vs. 0.51°, p = 0.02). After cyclic loading, ulnar-shaft rotation increased significantly in the plate–HCS group (0.97° to 1.16°, p = 0.02) but not in the double-plate group.

**Conclusion:**

In this cadaveric model, fixation with a palmar plate combined with an HCS provided comparable axial stability but demonstrated greater variability and less consistent rotational control compared with radiopalmar double plating. Clinical studies are required to determine whether this less invasive construct achieves equivalent outcomes in vivo.

## Introduction

Distal radius fractures are among the most common fractures in humans, representing the third most frequent cause of hospital admission in Germany in 2019 [1]. The incidence in Western populations is estimated at 200–300 per 100,000 inhabitants [2–4], with two characteristic peaks: young, athletic men and postmenopausal women over 50 years of age [5]. Anatomical reduction is essential, particularly in younger patients, to prevent post-traumatic arthrosis and to restore optimal wrist function [6]. In older, less active patients, minor residual deformities may be tolerated [7]. The introduction of palmar locking plates has substantially improved outcomes, even in osteoporotic bone [8, 9].

Palmar locking plates are the mainstay of surgical treatment for dorsally displaced fractures, providing high mechanical stability [10–12]. However, in complex intra-articular fracture patterns, palmar plating alone may be insufficient, leading to the use of combined fixation strategies [13]. Biomechanical studies have demonstrated superior stability of radiopalmar double plating compared with isolated palmar plates in AO/OTA 23-C2.1 fracture models [14].

The headless compression screw (HCS), originally developed for scaphoid fractures [15], has been successfully applied to other small bones, including the distal ulna [16] and radial head, often achieving results comparable to plate fixation [17]. The HCS provides direct interfragmentary compression. While its use in distal radius fractures is less established, early reports suggest potential advantages in stabilizing radial styloid or small articular fragments that are difficult to capture with a palmar plate [18]. In other anatomical sites, HCS fixation is associated with low complication rates [19], although some earlier studies reported increased complication risks [20, 21].

Given the proven efficacy of radiopalmar double plating for complex fractures, there is a clinical need for alternative constructs that are less invasive and technically less demanding while providing equivalent stability. Biomechanical data on the combination of a palmar plate with an HCS are limited. Considering that AO/OTA 23-C2.1 fractures account for a considerable proportion of distal radius fractures [22, 23], this study aims to compare the stability of a palmar plate–HCS construct with radiopalmar double plating.

We hypothesized that a palmar plate combined with an HCS would provide biomechanical stability comparable to double plating while being less invasive. To test this, we evaluated stiffness, bone mineral density, interfragmentary range of motion (ROM), and rotation (ROT) in a standardized human fracture model, and documented implant-related complications during instrumentation.

## Materials and methods

All experiments were conducted in the biomechanical laboratory of our university hospital. The protocol closely followed the methodology described by Jäger et al. [14].

### Specimens

Eleven matched pairs of cryopreserved human radii (stored at –24 °C) were obtained. Donors were aged 71–95 years (mean 84 years); eight were female and three male, all of whom had provided informed consent prior to death. No formal a priori power calculation was performed. The sample size was determined by specimen availability and is comparable to other cadaveric biomechanical studies in distal radius research. Before removal of soft tissues, the palmar and radial plates were temporarily positioned via the modified Henry approach [24] to assess fit and exposure. A radial approach was then performed to confirm implant positioning and inspect adjacent structures.

**Fig. 1:**
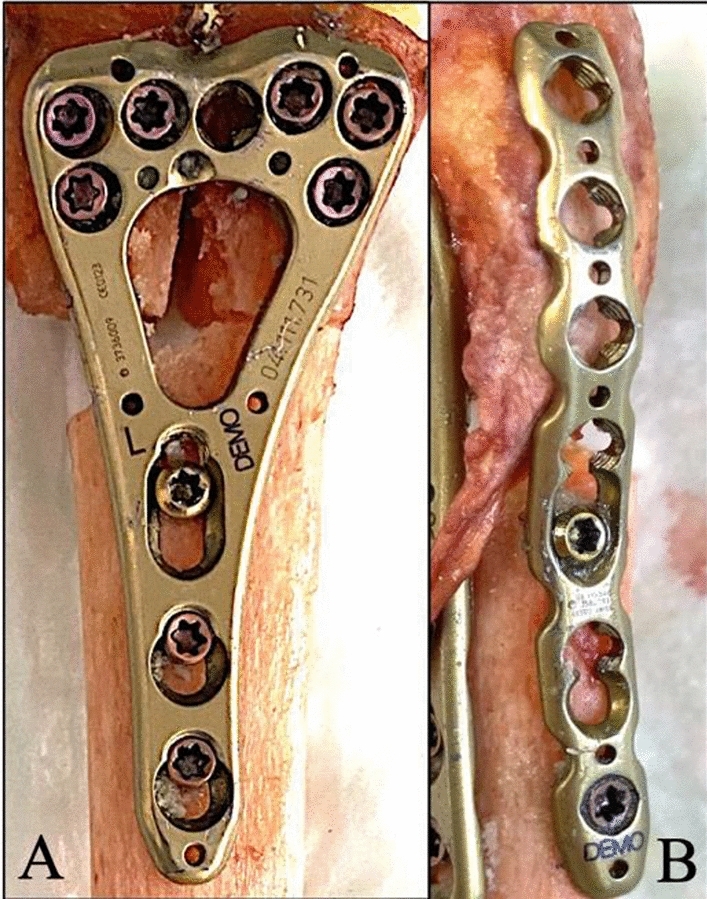
**A** Palmar plate on left distal radius with six distal locking screws, two proximal locking screws and one proximal cortex screw. **B** Radial plate on right distal radius secured with one cortex screw and one proximal locking screw. Own illustration

For biomechanical testing, the distal 12 cm of each radius was isolated, with all soft tissues removed except the brachioradialis tendon (see Fig. 1). Bone mineral density (BMD, mg/cm^3^) and T-scores were determined by quantitative computed tomography (qCT; GE Revolution EVO 64, GE Healthcare, Chicago, USA). Two pairs (pairs 5 and 49, both female) were excluded from analysis due to intra-test fracture or incomplete optical data acquisition.

### Fracture model

**Fig. 2:**
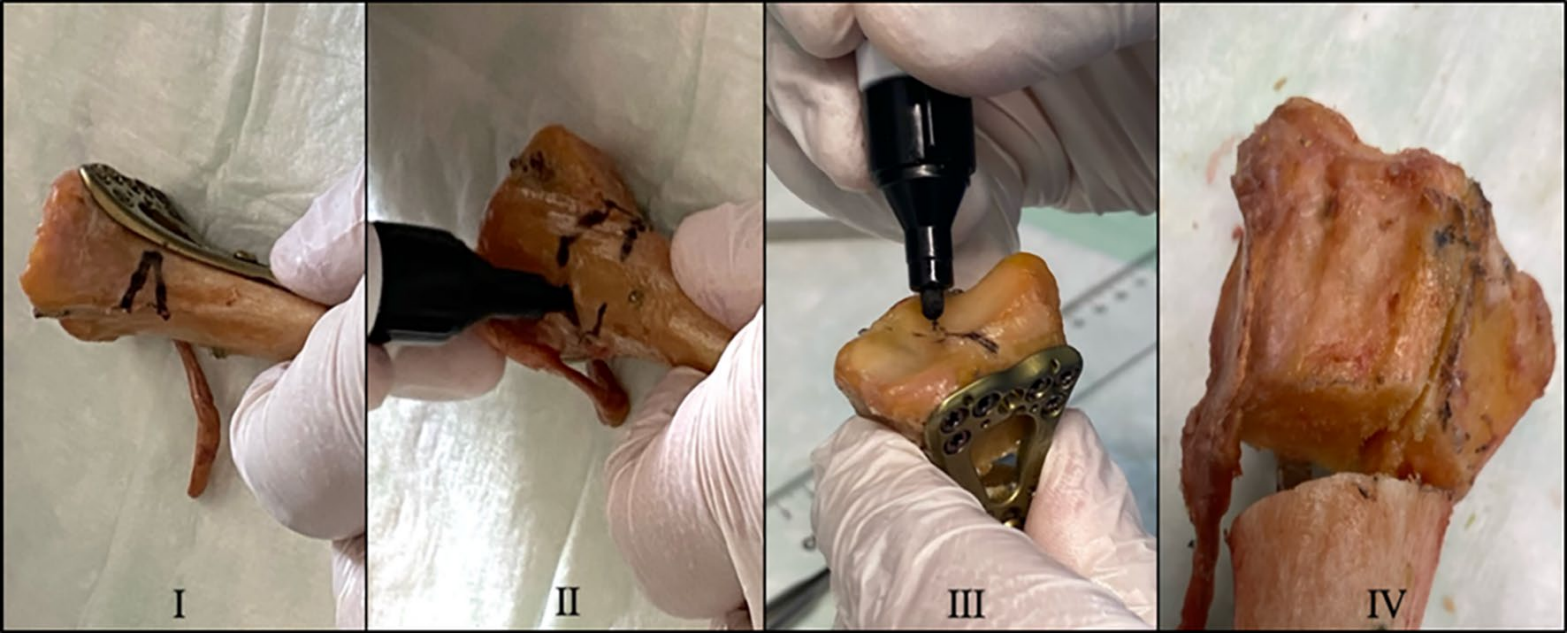
I/II: Ulnar and posterior view of distal radius specimen with marked 30° opening wedge, 2 cm proximal to the lunate fossa. III: Marked articular fracture component between scaphoid and lunate fossa. IV: Final fracture model with simple articular fracture and metaphyseal defect zone. Own illustration

A standardized AO/OTA 23-C2.1 fracture model was created to simulate a simple sagittal articular fracture with metaphyseal comminution (see Fig. 2). Fracture lines were marked on thawed bones and cut with a precision saw in accordance with previously published protocols [14].

### Implants and fixation techniques

Twenty-two radii were allocated as follows:

- Group 1 (left radii): palmar plate plus HCS
- Group 2 (right radii): radiopalmar double plate

The palmar fixation in both groups employed a 2.4 mm VA-LCP distal radius plate (DePuy Synthes, Zuchwil, Switzerland; see Fig. 1). Screws were inserted with standardized torque (0.8 Nm). For the HCS group, a 3.0 mm HCS with short thread (Zimmer Inc Warsaw (Indiana), USA) was placed radially to ulnarly under compression, ensuring that no threaded portion crossed the fracture gap. For the double-plate group, a 2.4 mm VA-LCP radial plate was positioned with one cortical and one locking screw proximally (see Fig. 1). The radial plate was inserted through a simulated modified Henry approach. Although soft tissue constraints were not mechanically limiting in this cadaveric setup (with preservation of the brachioradialis tendon only), distal screw placement was intentionally restricted to replicate the limited distal exposure typically achievable through this approach in vivo. The plate was therefore used primarily as a buttress construct, proximally fixed with two shaft screws. Slight overcontouring allowed the plate to exert compressive force from radial to ulnar, stabilizing the radial fragment against the ulnar fragment. This standardized approach was chosen to reflect clinical practice rather than to maximize construct rigidity under experimental conditions.

### Biomechanical testing

**Fig. 3:**
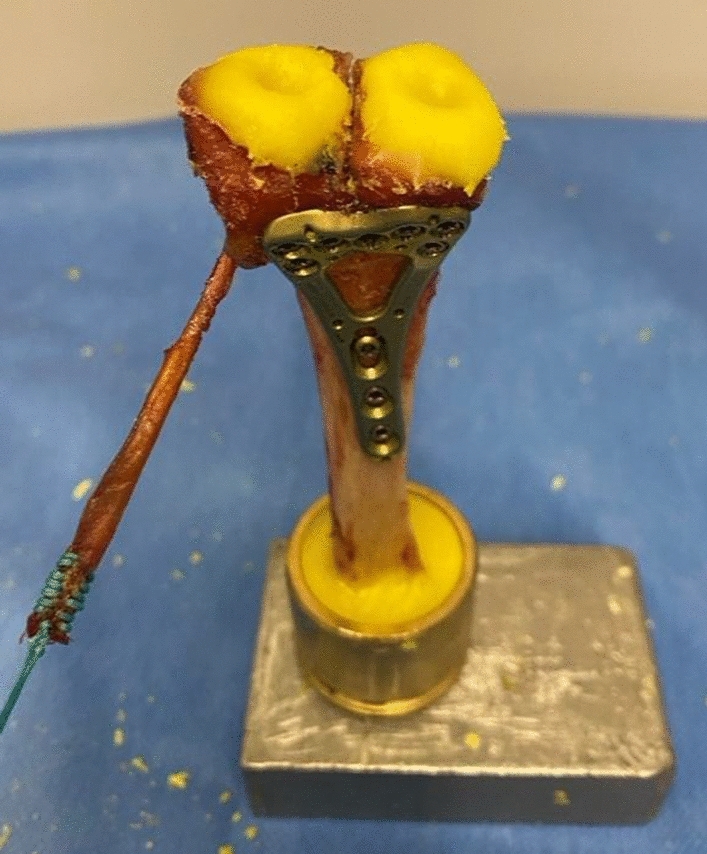
Embedded left radius with the proximal end fixed in a cylinder and oval recesses on the scaphoid and lunate fossae in preparation for biomechanical testing. On the left side of the image, the brachioradialis tendon is secured with Ethilon suture. Own illustration

**Fig. 4:**
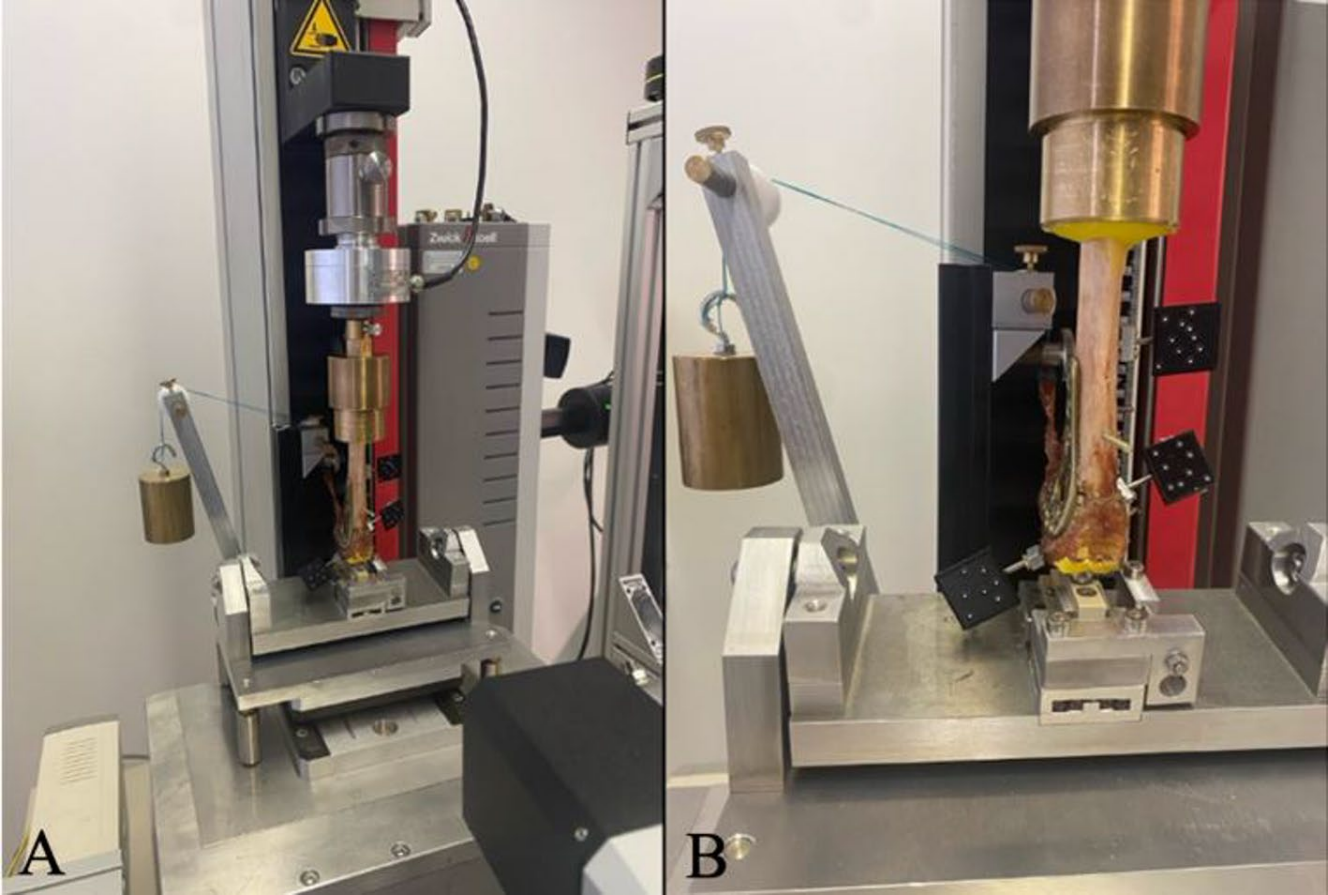
Universal testing machine Zwick 1.0 with the distal radius mounted. **A** Complete experimental setup including camera in the front. **B** Visible marker plates for camera motion tracking attached to different fragments. 500 g weight attached to brachioradialis tendon. Own illustration

Specimens were embedded in polymethylmethacrylate within custom aluminum fixtures (see Fig. 3). A constant 500 g load was applied to the brachioradialis tendon via suture loop. The proximal radius was mounted in a mechanical testing machine (Zwick1.0, ZwickRoell GmbH & Co. KG, Ulm, Germany), while the distal articular surface interacted with a rocker and metal spheres to simulate physiological load distribution (60% scaphoid fossa, 40% lunate fossa) (see Fig. 4) [25].

Relative fragment motions were recorded using an optical 3D motion-tracking system (Aramis 12 M, Carl Zeiss GOM Metrology GmbH, Braunschweig, Germany). The protocol comprised (1) static baseline testing, (2) cyclic axial loading at 150 N for 5000 cycles (0.2 Hz), and (3) post-cyclic static testing. Rotational displacement parameters were defined as fragment rotation occurring under axial compressive loading and were not intended to represent isolated torsional resistance of the constructs. This approach has been widely used in comparable distal radius biomechanical investigations to assess interfragmentary motion under physiologic axial loading conditions.

### Statistical analysis

Data were processed using SPSS (Version 28.0, IBM, Chicago, IL). Normality was assessed with the Kolmogorov–Smirnov test. As distributions were frequently non-normal, the Mann–Whitney U test was applied for intergroup comparisons and the Wilcoxon signed-rank test for paired analyses over time. Statistical significance was set at p < 0.05. Effect sizes were calculated according to Cohen (small: r > 0.1; medium: r > 0.3).

## Results

Two specimen pairs (nos. 5 and 49) were excluded due to intra-test fracture and insufficient optical data acquisition, leaving nine matched pairs for final analysis. Donor characteristics (age, sex, BMD, T-score) are summarized in Table 1.

**Table 1:** Bone mineral density and Stiffness in Plate-HCS vs. radio-palmar Double Plate Fixation

Parameter	Group 1 (Plate-HCS)	Group 2 (Double Plate)	p between	Entire cohort
Median Age (Years)	88 (81–89)	88 (81–89)	–	88 (81–88)
Sex (female/male)	6 / 3	6 / 3	–	12/6
Bone Mineral Density (mg/cm^3^)	107.6 (43.2–152.9)	120.0 (43.7–169.5)	0.73	113.8 (44.4–158.9)
T-value	−2.34 (−4.83–−0.90)	−1.86 (-4.79- -0.30)	0.73	−2.10 (-4.77- -0.68)
Initial Stiffness (N/mm)	187.1 (168.0–288.3)	142.9 (130.2–221.6)	0.19	180.3 (139.0–237.1)
Final Stiffness (N/mm)	197.3 (147.0–242.3)	141.0 (113.8–205.5)	0.14	166.3 (128–232.5)
Change in Stiffness	p = 0.26	p = 0.37		p = 0.17

Metric values are presented as median with interquartile range (IQR)

p-values of comparison between groups using Mann–Whitney-U-Test; p-values of comparison between two time points within groups using Wilcoxon-signed-rank-Test

### Stiffness

Stiffness did not differ significantly between groups, either before or after cyclic loading. Both constructs maintained comparable values with no evidence of implant loosening or mechanical failure (see Table 1).

### Axial displacement

Axial ROM and displacement were similar in both groups at baseline and after cyclic loading, with no statistically significant differences (see Table 2).

**Table 2:** Range of Motion in Plate-HCS vs. radio-palmar Double Plate Fixation

ROM Parameter	Measurement	Group 1 (Plate-HCS)	Group 2 (Double Plate)	p between	Entire cohort
Axial Displacement (µm)	Initial	802 (524–896)	1050 (679–1157)	0.19	834 (635–1084)
	Final	761 (620–1028)	1066 (744–1316)	0.14	904 (646–1174)
	p within	0.48	0.95		0.57
Radial-Shaft (µm)	Initial	439 (157–777)	250 (160–362)	0.19	280 (169–491)
	Final	330 (135–773)	215 (130–283)	0.19	248 (142–488)
	p within	0.77	0.67		0.98
Ulnar-Shaft (µm)	Initial	130 (80–220)	153 (111–251)	0.54	134 (98–222)
	Final	145 (100–180)	200 (113–263)	0.39	155 (103–228)
	p within	0.26	0.40		0.18
Radial-Ulnar (µm)	Initial	40 (23–109)	59 (51–65)	0.23	58 (35–65)
	Final	47 (33–123)	75 (50–105)	0.40	54 (35–108)
	p within	0.92	0.40		0.39

Metric values are presented as median with interquartile range (IQR)

p-values of comparison between groups using Mann–Whitney-U-Test; p-values of comparison between two time points within groups using Wilcoxon-signed-rank-Test

### Interfragmentary motion (ROM, ROT)

Interfragmentary ROM did not differ significantly between groups. However, variability was greater in the plate–HCS group, with wider ranges of radial and ulnar shaft motion (see Table 2).

**Fig. 5:**
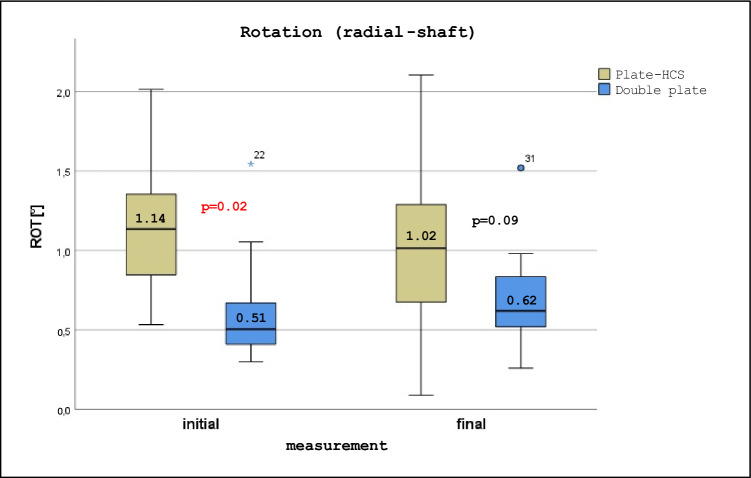
Boxplots of rotation between radial articular fragment and shaft fragment within Plate-HCS and double plate groups at initial and final measurement in degrees. p < 0.05 highlighted in red

Radial shaft rotation was significantly greater in the plate–HCS group at baseline (p = 0.02; see Fig. 5), but this difference was no longer present after cyclic loading. Ulnar shaft rotation increased significantly after cyclic loading in the plate–HCS group (p = 0.02), whereas no significant change was observed in the double-plate group (see Table 3).

**Table 3:** Rotation in Plate-HCS vs. radio-palmar Double Plate Fixation

ROT Parameter	Measurement	Group 1 (Plate-HCS)	Group 2 (Double Plate)	p between	Entire cohort
Radial-Shaft (°)	Initial	1.14 (0.77–1.37)	0.51 (0.41–0.86)	**0.02**	0.69 (0.49–1.24)
	Final	1.02 (0.62–1.45)	0.62 (0.49–0.91)	0.09	0.84 (0.55–1.26)
	p within	0.56	0.37		0.29
Ulnar-Shaft (°)	Initial	0.97 (0.44–1.27)	0.59 (0.18–0.77)	0.17	0.71 (0.40–1.15)
	Final	1.16 (0.56–1.36)	0.70 (0.57–1.05)	0.55	0.76 (0.57–1.31)
	p within	**0.02**	0.16		** < 0.01**
Radial-Ulnar (°)	Initial	0.32 (0.17–0.87)	0.31 (0.23–0.67)	0.85	0.29 (0.20–0.42)
	Final	0.16 (0.14–0.36)	0.38 (0.21–0.43)	0.20	0.34 (0.17–0.43)
	p within	0.17	0.21		0.06

Metric values are presented as median with interquartile range (IQR)

p-values of comparison between groups using Mann–Whitney-U-Test; p-values of comparison between two time points within groups using Wilcoxon-signed-rank-Test; numbers in bold indicating statistical significance with p<0.05

### Complications

No implant breakage, screw loosening, or catastrophic failure occurred in either group during cyclic loading. One intraoperative complication—tendon entrapment during radial plate insertion—was observed in the double-plate group.

## Discussion

This study compared the biomechanical stability of two fixation methods for AO/OTA 23-C2.1 distal radius fractures with metaphyseal defect zones: a palmar plate combined with a headless compression screw (HCS) versus radiopalmar double plating. Both constructs achieved comparable stability regarding stiffness, axial displacement, and interfragmentary ROM, with no evidence of implant loosening or mechanical failure. However, differences in rotational behavior were observed.

Initial radial shaft rotation was significantly higher in the plate–HCS group compared with double plating, although this difference disappeared after cyclic loading. In contrast, ulnar shaft rotation increased significantly after cyclic loading in the plate–HCS group but remained stable in the double-plate group. Taken together, these findings indicate that while both fixation strategies provide sufficient overall stability, radiopalmar double plating offers more consistent rotational control, whereas the plate–HCS construct is associated with greater variability.

Our results are consistent with previous biomechanical studies demonstrating the superior stability of radiopalmar double plating compared with isolated palmar plating in complex distal radius fractures [14]. They expand upon this evidence by showing that supplementing a palmar plate with an HCS can approximate the biomechanical stability of double plating. The HCS provides direct interfragmentary compression and may be particularly advantageous for stabilizing radial styloid fragments that are difficult to adequately capture with a palmar plate alone [18]. Comparable biomechanical performance and complication rates of HCS fixation have been reported for scaphoid [26, 27] and metacarpal fractures [19], although increased complication rates have been described in certain anatomical regions [20, 21].

From a clinical perspective, the plate–HCS construct may offer potential advantages. Although radiopalmar double plating can be performed through the same modified Henry approach, the addition of a second plate has been associated with tendon irritation and extensor tendon complications [28–31]. In contrast, augmenting a palmar plate with an HCS is technically less demanding and may reduce the risk of soft-tissue irritation. Particularly in osteoporotic bone, this approach could provide sufficient fixation while limiting surgical morbidity. However, the increased variability observed in rotational stability underscores the importance of meticulous intraoperative screw placement and fracture compression when using an HCS.

Several limitations should be considered. The cadaveric specimens were derived from elderly donors with reduced bone quality, which may limit generalizability to younger or more active patient populations. The standardized fracture model, while well established [14, 32, 33], does not fully reproduce the heterogeneity of clinical fracture patterns or the influence of surrounding soft tissues. Furthermore, loading conditions were simplified, with 5000 cycles at 150 N of only axial loading. No isolated torsional loading, combined loading scenarios, or load-to-failure testing were performed. Consequently, rotational stability was assessed indirectly under axial conditions and may not fully reflect behaviour under physiological multidirectional wrist loading [34]. Finally, the relatively small sample size, although comparable to other biomechanical studies, may increase the risk of type II error, particularly regarding rotational parameters with high variability. Therefore, absence of statistically significant differences should not be interpreted as proof of equivalence of constructs.

## Conclusion

In this cadaveric model, a palmar plate combined with a headless compression screw provided comparable axial stability but demonstrated greater variability and less consistent rotational control compared with radiopalmar double plating for AO/OTA 23-C2.1 distal radius fractures. Both constructs resisted axial displacement and maintained stiffness under cyclic loading; however, radiopalmar double plating demonstrated more consistent rotational control, whereas the plate–HCS construct exhibited greater variability.

The plate–HCS technique represents a less invasive and technically simpler alternative, but its reproducibility and rotational reliability require further investigation. Prospective clinical studies are needed to determine whether this approach achieves comparable safety, early mobilization, and long-term functional outcomes in vivo.

## Data Availability

The data presented in this study are not publicly available but available on request from the corresponding author. The data are not publicly available due to privacy and ethical restrictions.

## Abbreviations

AO: Arbeitsgemeinschaft für Osteosynthesefragen (Association for the Study of Internal Fixation)
HCS: Headless compression screw
ROM: Range of motion
ROT: Rotation
qCT: Quantitative computed tomography
M.: Musculus
VA-LCP: Variable angle locking compression plate
LCP: Locking compression plate
IQR: Inter quartile range
Nm: Newton meter
µm: Micrometer
N/mm: Newton per millimeter (unit of stiffness)
